# CALENDAR

**DOI:** 10.1054/bjoc.2000.1661

**Published:** 2001-02


					5-6 March 2001
Second International Conference on Cancer and the
Adolescent
Royal College of Physicians, London, UK
Further information from:
Conference Administrator, Samantha Greshoff. Tel: +44 (0) 20
8333 6311; Fax: +44 (0) 20 8856 3017;
E-mail: sam@greshoff.free-online.co.uk
13-14 March 2001
National Cancer Nursing Conference
Commonwealth Institute,
London, UK
Further information from:
Conference office, Emap Healthcare Events, Greater London
House, Hampstead Road, London NW1 7EJ, UK.
Tel: +44(0) 20 7874 0294; E-mail: conference@healthcare.emap.
co.uk; Website: http://www.nursingtimes.net/shop/shop_index.asp
24-26 May 2001
First International Symposium on Target Volume
Definition in Radiation Oncology
Limburg/Lahn, Germany
Further information from:
Assoc. Prof. Dr IC Kiricuta, Institute for Radiation Oncology,
St. Vincenz-Hospital, Limburg/Lahn, Germany.
Tel: +49 6431 292 4598; Fax: +49 6431 292 4163
1-4 July 2001
British Cancer Research Meeting 2001
University of Leeds, UK
Closing date for abstract submission: 16 February 2001
Further information from:
BCRM Secretariat, c/o Institute of Cancer Research,
Cotswold Road, Sutton, Surrey SM2 5NG, UK.
Tel: +44(0) 20 8722 4208; Fax: +44(0) 20 8770 1395;
E-mail: bcrm@icr.ac.uk
National Cancer Institute
Alternative Medicine in Cancer: Opening Doors to Research
NCI's Best Case Series Program
Is your complementary or alternative medicine (CAM) helping
cancer patients? NCI invites you to submit data from your best
cases for review by conventional research and CAM experts.
For details or a submission package, contact:
Office of Cancer Complementary and Alternative Medicine,
National Cancer Institute, NIH, Executive Plaza North,
Suite 102, Bethesda, MD 20892, USA. Tel: +1 (301) 435 7980;
Fax: +1 (301) 480 7980; E-mail: ncioccam-r@mail.nih.gov;
Website: http://occam.nci.nih.gov
CALENDAR
British Journal of Cancer (2001) 84(3), 442
(c) 2001 Cancer Research Campaign
doi: 10.1054/ bjoc.2000.1661, available online at http://www.idealibrary.com on
442

				

